# Increase expression of CD177 in Kawasaki disease

**DOI:** 10.1186/s12969-019-0315-8

**Published:** 2019-04-03

**Authors:** Ying-Hsien Huang, Mao-Hung Lo, Xin-Yuan Cai, Shih-Feng Liu, Ho-Chang Kuo

**Affiliations:** 1 Department of Pediatrics, Kaohsiung Chang Gung Memorial Hospital and Chang Gung University College of Medicine, #123 Da-Pei Road, Niaosong District, Kaohsiung, 83301 Taiwan; 2 Kawasaki Disease Center, Kaohsiung Chang Gung Memorial Hospital, #123 Da-Pei Road, Niaosong District, Kaohsiung, 83301 Taiwan; 3 Division of Pulmonary & Critical Care Medicine, Department of Internal Medicine, Kaohsiung Chang Gung Memorial Hospital and Chang Gung University College of Medicine, Kaohsiung, Taiwan; 4 Department of Respiratory Therapy, Kaohsiung Chang Gung Memorial Hospital and Chang Gung University College of Medicine, Kaohsiung, Taiwan

**Keywords:** Kawasaki disease, CD177, Intravenous immunoglobulin resistance

## Abstract

**Background:**

Kawasaki disease (KD) is the most common acute coronary vasculitis disease to occur in children. Its incidence has been attributed to the combined effects of infection, genetics, and immunity. Although the etiopathogenesis of KD remains unknown, we have performed a survey of global genetic DNA methylation status and transcripts expression in KD patients in order to determine their contribution to the pathogenesis of KD.

**Methods:**

We recruited 148 participants for this case-control study. The chip studies consisted of 18 KD patients that were analyzed both before undergoing intravenous immunoglobulin (IVIG) treatment and at least 3 weeks afterward, as well as 36 non-KD control subjects, using Illumina HumanMethylation450 BeadChip and Affymetrix GeneChip® Human Transcriptome Array 2.0. We then carried out real-time quantitative PCR on a separate cohort of 94 subjects for validation.

**Results:**

According to our microarray study, CD177, a neutrophil surface molecule, appeared to be significantly upregulated in KD patients when compared to controls with epigenetic hypomethylation. After patients received IVIG treatment, CD177 mRNA levels decreased significantly. PCR validation indicated that the CD177 expression is consistent with the Transcriptome Array 2.0 results. Furthermore, the area under the curve values of CD177 between KD patients and controls is 0.937. We also observed significantly higher CD177 levels in typical KD than in incomplete presentation or KD with IVIG resistance.

**Conclusion:**

In this study, we have demonstrated the epigenetic hypomethylation and increased expression of CD177 during the acute stage of KD. Furthermore, a higher expression of CD177 in KD patients with typical presentation was associated with IVIG resistance.

## Background

Kawasaki disease (KD) is an acute-onset systemic vasculitis of medium-sized vessels that affects multiple systems. It has an unknown etiology and primarily affects children under the age of 5 years old [1]. We acknowledge Tomisaku Kawasaki first described 50 cases of KD in the English language in 1974 [2]. Physicians diagnose KD with the following clinical symptoms: prolonged fever for more than five days, conjunctivitis, diffuse mucosal inflammation, polymorphous skin rashes, indurative edema of the hands and feet associated with peeling of the finger tips, and nonsuppurative lymphadenopathy [3]. However, 20–30% of KD patients do not completely fulfill the diagnostic criteria and are thus considered as having incomplete KD, which often makes diagnosis challenging for clinicians [4, 5]. Currently, no single laboratory examination can serve as the golden standard diagnostic tool for KD. The vascular involvement of KD occurs in small and medium-sized blood vessels, particularly coronary arteries [4]. The most severe complication of KD is coronary artery aneurysm (CAA), which approximately 20% of untreated children develop [6]. Therefore, KD is a primary cause of acquired heart disease in children in developed countries [7]. Although the etiopathogenesis of KD remains unknown, growing evidence has demonstrated that KD may be the result of a combination of infection, genetics, and immunity [8]. Furthermore, while many inflammatory mediators have been found to be elevated in the peripheral blood of KD patients, no biomarker can yet effectively predict the susceptibility, morbidity, prognosis, and treatment response of KD, and the susceptible gene(s) and immunopathogenesis still need to be clarified.

Epigenetics describes the acetylation pattern of the genome and DNA methylation and subsequently changes the chromatin structure [9]. In short, DNA methylation can deactivate genes [10]; meanwhile, demethylation changes of CpG sites indicate a contradictory change in gene expression [11]. In previous studies, we found that IVIG treatment significantly altered methylation patterns in KD patients [12–14], as well as that KD patients demonstrated considerably increased mRNA expression in toll-like receptors (TLRs) and hypomethylation at the gene promoters of TLRs [15]. IVIG treatment can restore the methylation level of TLRs and decrease their mRNA expression [15]. We were also the first to report epigenetic hypomethylation, increased transcripts, and the upregulation of NLRC4, NLRP12, and IL-1β in KD patients [16]. In general, KD-induced inflammasomes can sense innate immune system receptors and sensors in response to external infectious microbes or host protein molecules [17].

In light of the distinct genetic and epigenetic differences of KD, we adopted Illumina HumanMethylation450 BeadChip and Affymetrix GeneChip® Human Transcriptome Array 2.0 to assess their CpG markers and expression levels and comprehensively compare the genetic expressions of KD patients to control subjects. The purpose of this study was to identify novel targets with a clinical significance and potential prognostic value for KD patients.

## Methods

### Patients

To be included in this study, KD patients were required to meet the KD diagnosis criteria stipulated by the American Heart Association [18, 19] and had to receive a single high-dose IVIG treatment (2 g/kg) over 12 h in our hospital. Patients that presented with fewer than four of the five principal findings of KD were considered as having incomplete (atypical) KD [20]. In this case-control study, we utilized Illumina HumanMethylation450 BeadChip and Affymetrix GeneChip® Human Transcriptome Array 2.0 to quantify and compare genetic methylation and transcript expressions of genes in 18 KD patients (both before and at least 3 weeks after IVIG treatment), as well as in 18 healthy and 18 febrile controls. We then validated the mRNA levels of genes in 46 KD patients, 24 healthy controls, and 24 febrile subjects using real-time quantitative PCR. The baseline demographics are listed in Table 1. The subjects in the fever control group were diagnosed with acute pharyngitis, tonsillitis, bronchopneumonia, pneumonia, or urinary tract infection. Furthermore, we collected peripheral blood samples from KD patients both before undergoing IVIG treatment (pre-IVIG) and then again at least 3 weeks after completing IVIG treatment, as previously described in another study [21]. We defined CAL as a coronary artery with an internal diameter of at least 3 mm (4 mm if the patient was older than 5 years) or a segment with an internal diameter at least 1.5 times larger than that of an adjacent segment, as identified through echocardiography [22, 23]. IVIG resistance was defined as no defervescence (defined as a temperature > 38 °C) 48 h after the completion of IVIG treatment or fever recurrence 7 days after IVIG treatment with marked inflammation signs as in our previous reports [23, 24]. This study was approved by the Chang Gung Memorial Hospital’s Institutional Review Board, and we obtained written informed consent from the parents or guardians of all participants. Enrolled children were permitted to withdraw from the study at any time during the study period, and we anonymized all experimental results prior to analysis.

**Table 1:** Baseline characteristics of patients with KD and controls

Characteristic	Healthy controls *n* = 24	Febrile controls *n* = 24	KD (complete/incomplete) *n* = 46 (36/10)	*P*-value
Male gender	14	15	35 (26/9)	0.324
Age (y)	7.5 ± 1.0^a^	2.6 ± 0.3^b^	1.6 ± 0.2^b^ (1.6 ± 0.2/1.7 ± 0.9)	< 0.001
Age range (y)	0–16	0–5	0–9 (0–5/0–9)	
Fever duration (day)		5.1 ± 0.6	6.7 ± 3.4 (6.0 ± 0.4/9.1 ± 1.7)	0.06
WBC (1000/uL)	8.0 ± 0.6^a^	9.3 ± 1.0^a^	13.7 ± 0.7^b^ (13.6 ± 0.8/14.4 ± 1.3)	< 0.001
RBC (million/Ul)	4.9 ± 0.1^a^	4.7 ± 0.1^a^	4.3 ± 0.1^b^ (4.3 ± 0.1/4.3 ± 0.1)	< 0.001
Hemoglobin (g/dL)	12.7 ± 0.2^a^	12.2 ± 0.2^a^	11.0 ± 0.1^b^ (11.1 ± 0.2/10.5 ± 0.2)	< 0.001
CRP (mg/L)		23.5 ± 4.5^a^	99.2 ± 11.1^b^ (104.4 ± 12.9/ 80.5 ± 21.5)	< 0.001
CAL formation			22 (15/7)	
IVIG resistance			8 (8/ 0)	

*CAL* coronary artery lesion, *IVIG* intravenous immunoglobulin, *KD* Kawasaki disease. Data expressed as mean ± SEM

a vs b indicates siginifcant difference

b vs b indicates no significant difference

^a,b^ Significantly important

### Experiment design

To carry out this study, we collected whole blood samples from all of the participants and submitted them to white blood cell (WBC) enrichment, as we have already described in previous studies [12, 13].

### DNA methylation profiling with Illumina M450K BeadChip

We utilized Illumina HumanMethylation450 (M450K) BeadChip to perform genome-wide screening of DNA methylation patterns. The M450K BeadChip program was created to detect methylation patterns of approximately 450,000 CpG markers, thus spanning the entire human genome. We applied 200 ng of bisulfite-converted genomic DNA to each M450K BeadChip assay in accordance with the manufacturer’s instructions [12]. Then, we calculated the methylation percentage of cytosine for each CpG marker in each sample, which we referred to as the β value. The resulting raw data were analyzed with Partek [14]. All DNA methylation data were submitted to NCBI GEO; please refer to GSE109430 for further information [14].

### Gene expression profiling with microarray

To obtain unbiased results, we created four pooled RNA libraries by evenly pooling six RNA samples, which resulted in three pooled healthy control, three fever control, three pre-IVIG, and three post-IVIG libraries, as previously described [15]. We performed microarray assay on the pooled RNA samples to establish gene expression profiles and then also carried out profiling with GeneChip® Human Transcriptome Array 2.0 (HTA 2.0, Affymetrix, Santa Clara). We measured the RNA concentrations with the NanoDrop 2000 spectrophotometer (Thermo Scientific, MA, USA). All RNA samples passed the criterion of a RIN ≥ 7 when assessed with the Agilent 2100 Bioanalyzer (Agilent, CA, USA) as described in a previous study [14]. We prepared the RNA samples with the WT PLUS Reagent kit and performed hybridization on the HTA 2.0 microarray chips. Pursuant to the Affymetrix instruction manual, we subjected the HTA 2.0 chips’ raw data to quality control examination and then analyzed the data with Partek [14]. All microarray data were submitted to NCBI GEO; please refer to GSE109351 for more information [14].

### RNA isolation and real-time quantitative RT-PCR

To quantify the mRNA levels of CD177, we selected the LightCycler® 480 Real-Time PCR System (Roche Molecular Systems, Inc., IN, USA) to carry out real-time quantitative PCR. We separated the total mRNA from the WBC with an isolation kit (mirVana™ miRNA Isolation Kit, Catalog number: AM1560, Life Technologies, Carlsbad, CA) and then calculated both the quality (RIN value) and quantity of the RNA samples using Bioanalyzer (ABI) and Qubit (Thermo), respectively, according to the manufacturers’ instructions. All RNA samples passed the criterion of RIN≧7. We performed PCR using a SYBR Green PCR Master Mix containing 10 μM of specific forward and reverse primers. Furthermore, we carried out the relative quantification of gene expression using the comparative threshold cycle (C_T_) method, which allowed us to determine the target amount as 2^−(ΔCT target − Δ CT calibrator)^ or 2^−ΔΔCT^ [25]. Primers were designed to amplify CD177 and RNA18S5 (internal control) as forward 5′- CTGGTTCACGTCTCCAAACC-3′ and reverse 5′- TCTCCTGCAGTTGCTCAGAT -3′, as well as forward 5′- GTAACCCGTTGAACCCCATT-3′ and reverse 5′-CCATCCAATCGGTAGTAGCG-3′, respectively. All experiments were done twice to verify and validate the amplification efficiencies.

### Statistical analysis

All data are presented as mean ± standard error. Once chips passed the quality control criteria, we evaluated them using Partek (Partek, St. Louis), a commercial software specifically designed to analyze microarray data. We adopted one-way ANOVA or Student’s t-test as appropriate to evaluate the quantitative data and paired sample *t-*test to evaluate any data changes before and after undergoing IVIG treatment [15]. The receiver operating characteristics curve method was used to differentiate between groups. We carried out all statistical analyses with SPSS version 12.0 for Windows XP (SPSS, Inc., Chicago, USA), and a two-sided *p*-value less than 0.05 was considered statistically significant.

## Results

### Epigenetic hypomethylation and upregulated CD177 mRNA levels in KD patients compared to controls and changes following IVIG treatment

**Fig. 1:**
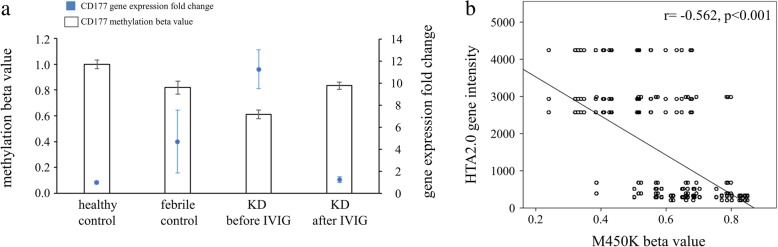
**a**. Integration of CpG marker methylation patterns and gene expression profiles of CD177. The methylation patterns of the representative CpG marker (cg22537604) and gene expression profiles showed negative tendencies and were observed to change in both the healthy and febrile control subjects, as well as Kawasaki disease (KD) patients before and after undergoing intravenous immunoglobulin treatment. The histogram and curve are presented as mean ± standard error. **b**. We adopted scatter plots to represent the relationship between mRNA levels and DNA methylation, which demonstrate that mRNA levels were negatively correlated with DNA methylation (Pearson’s correlation coefficient r = − 0.562, *p* < 0.001)

In this study, we focused on the variations in genetic profiles between control subjects and KD patients. DNA methylation has traditionally been believed to be negatively correlated with gene expression [26]. The lower a CpG marker was methylated, the more abundantly the gene was expressed [26]. In the beginning, we chose the ideal genes for possessing a negative correlation with CpG markers with a 5% change in M450K and a two-fold change in HTA 2.0 between KD patients and controls. The 14 genes that we identified are listed in Table 2. The majority of these genes were glycoprotein (7/14) and involved in innate immunity (4/14) and neutrophil migration (3/14). We observed that CD177 demonstrated the most significant fold change between healthy (11.263 folds, *p* = 0.001) and febrile controls (3.429 folds, *p* = 0.032) among these 14 genes. As shown in Fig. 1, the mRNA levels of CD177 were significantly higher in KD patients than in the control groups. The CD177 values in KD patients decreased significantly after undergoing IVIG treatment. Furthermore, the mRNA expression level and DNA methylation (cg22537604) of CD177 have a negative correlation in both KD patients and controls (Pearson’s correlation coefficient r = − 0.562, *p* < 0.001).

**Table 2:** The ideal genes for possessing negative correlation with CpG markers with a 5% change in M450K and a two-fold change in HTA 2.0 between KD patients and controls

Symbol	Annotated Term	RefSeq	Fold-Change (KD1 vs. HC)	*p*-value (KD1 vs. HC)	Fold-Change (KD1 vs. FC)	*p*-value (KD1 vs. FC)	Fold-Change (KD3 vs. KD1)	*p*-value (KD3 vs. KD1)
CD177	Glycoprotein, Neutrophil migration	NM_020406	11.263	0.001*	3.429	0.032*	−9.022	0.002*
VNN1	Glycoprotein	NM_004666	4.850	0.000*	2.508	0.000*	−5.969	0.000*
NLRC4	Apoptosis, Innate immunity	NM_001199138	4.454	0.000*	2.159	0.015*	−4.761	0.000*
S100A12	Antibiotic, Innate immunity, Neutrophil migration	NM_005621	3.899	0.001*	2.037	0.033*	−4.524	0.001*
GPR84	Glycoprotein	NM_020370	3.821	0.004*	2.266	0.041*	−4.167	0.003*
ARG1	Arginase activity, Manganese ion binding	NM_000045	3.735	0.001*	2.020	0.019*	−4.083	0.000*
CLEC4D	Glycoprotein, Innate immunity	NM_080387	3.684	0.008*	2.873	0.022*	−4.474	0.004*
HP	Glycoprotein, Immunity	NM_001126102	3.681	0.000*	2.387	0.005*	−3.993	0.000*
EMR1	G-protein coupled receptor activity	NM_001256252	3.610	0.002*	2.285	0.017*	−2.822	0.006*
CR1	Innate immunity, Glycoprotein	NM_000573	2.885	0.001*	2.333	0.004*	−2.915	0.001*
P2RY14	Glycoprotein	NM_001081455	2.851	0.005*	2.266	0.017*	−2.636	0.008*
CLC	Carbohydrate binding	NM_001828	2.754	0.018*	2.482	0.029*	−2.065	0.067
LPCAT2	Calcium ion binding	NM_017839	2.116	0.009*	2.227	0.006*	−2.070	0.010*
IL1B	Inflammatory response, Neutrophil migration	NM_000576	1.677	0.066	2.195	0.012*	−2.475	0.006*

*KD1* Kawasaki disease before IVIG treatment, *KD3* Kawasaki disease > 3 weeks after IVIG treatment, *FC* febrile control, *HC* healthy control

*indicate *p* value < 0.05

### CD177 expressions in the peripheral white blood cells (WBCs) of KD patients and controls

**Fig. 2:**
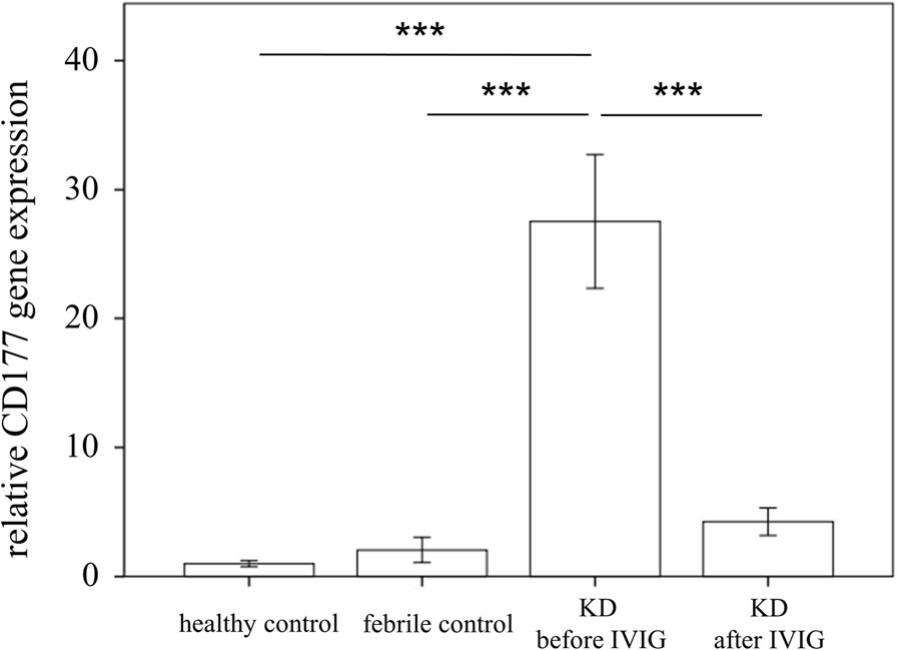
Analyses of CD177 mRNA in the peripheral white blood cells of Kawasaki disease (KD) patients (n = 46) and healthy and febrile controls (n = 24, respectively) using a real-time quantitative polymerase chain reaction. Data are expressed as mean ± standard error. IVIG, intravenous immunoglobin, ***indicates *p* < 0.001 between the groups

**Fig. 3:**
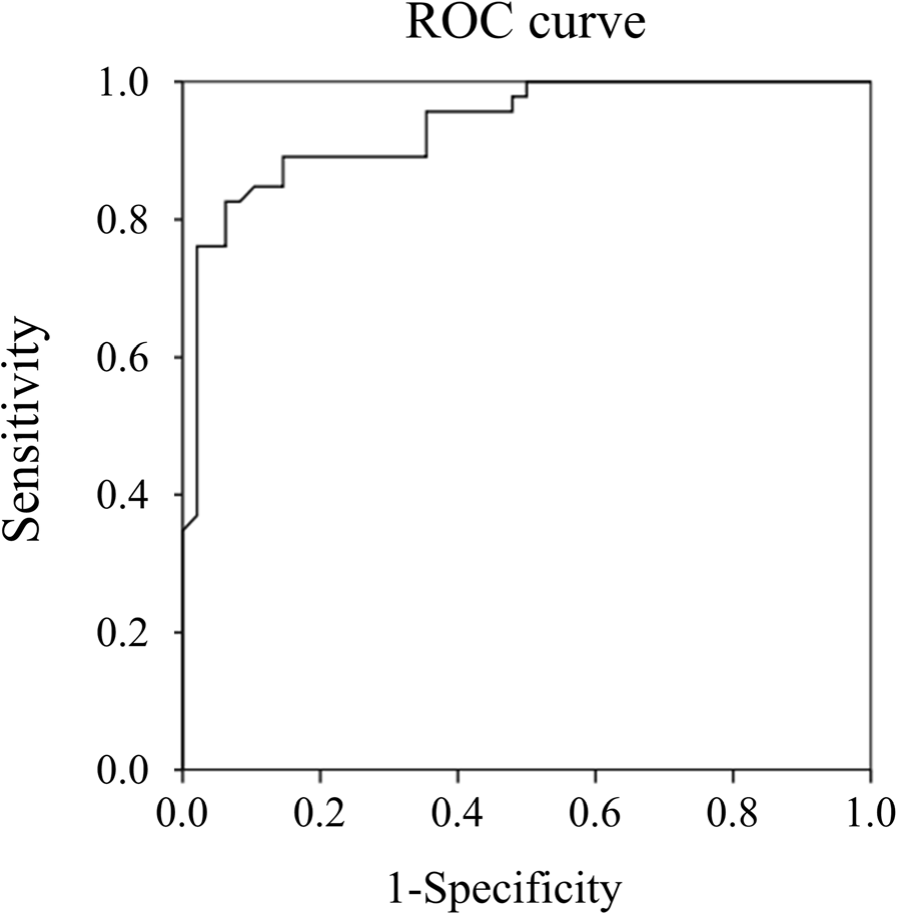
The area under the curve (AUC) of mRNA levels of CD177. The AUC value of CD177 between Kawasaki disease (KD) patients (*n* = 46) and controls (*n* = 48) is 0.937, with a 95% confidence interval from 0.89 to 0.98

**Fig. 4:**
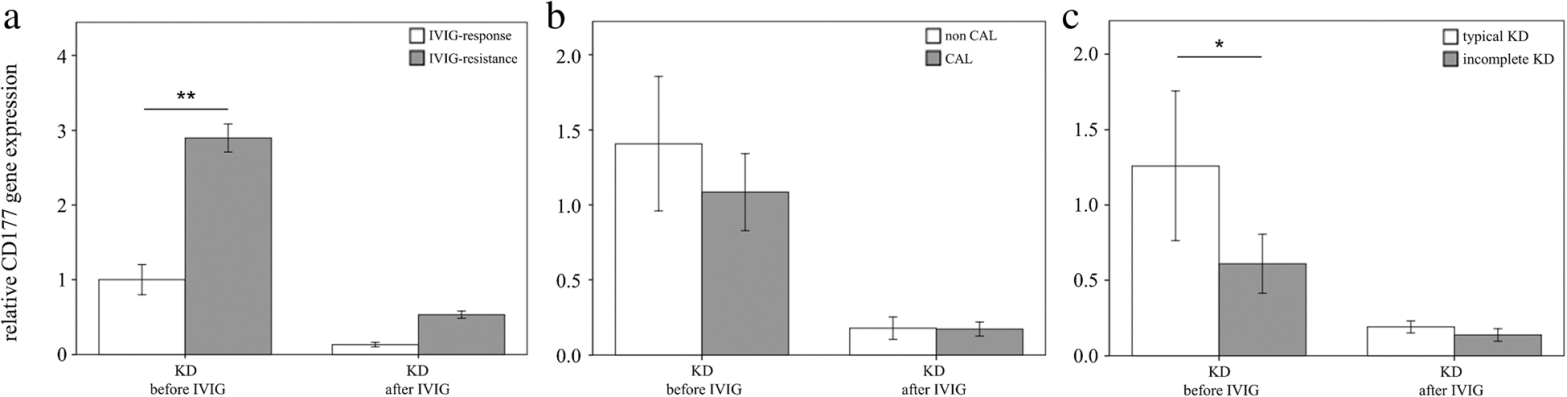
Analyses of CD177 mRNA in the peripheral white blood cells of Kawasaki disease (KD) patients (*n* = 46) using a real-time quantitative polymerase chain reaction between patients with KD **a**. without (*n* = 24) and with (*n* = 22) coronary artery lesions (CAL), **b**. without (*n* = 38) and with (*n* = 8) intravenous immunoglobulin resistance, and **c**. typical (*n* = 36) and incomplete (*n* = 10) presentation before IVIG treatment and after undergoing IVIG treatment. *p < 0.05. Data are presented as mean ± standard error

We conducted qPCR assays on CD177 to investigate the mRNA levels of CD177 in a separate cohort of 46 KD patients, 24 febrile controls, and 24 healthy controls (Table 1). We observed no significant difference between age and fever duration between febrile controls and KD patients. Our results showed elevated CD177 in KD patients compared to the controls, as well as that CD177 considerably decreased 3 weeks after IVIG treatment (all *p* < 0.001, Fig. 2). Moreover, we observed no significant association between CD177 and WBC or CRP (*p* = 0.729 and 0.493, respectively) in multiple linear regression analysis. These findings agree with the results of HTA 2.0. Furthermore, the area under the curve (AUC) values of CD177 between KD patients and controls was 0.937, with a 95% confidence interval from 0.89 to 0.98 (Fig. 3). We also observed significantly higher CD177 mRNA levels in KD patients with IVIG resistance prior to IVIG treatment (*p* = 0.003, Fig. 4a). However, we found no significant difference in the CD177 mRNA levels between KD patients with CAL and those without (Fig. 4b). We further compared the mRNA level between patients with complete KD syndromes (typical) and those without (incomplete). Interestingly, the CD177 mRNA levels were higher in the complete KD patients than in the incomplete KD patients (*p* = 0.016) (Fig. 4c).

## Discussion

While growing evidence has indicated that cytokine profiles are associated with the pathogenesis of KD, the precise immunopathogenesis of Kawasaki disease is still unclear. Our noteworthy observations include the epigenetic hypomethylation and upregulation of CD177 in KD, as well as increasing mRNA levels of CD177 in KD patients and their association with IVIG resistance. We have also related higher CD177 levels more to typical KD presentation than incomplete KD.

In our recent study of integrating genome-wide DNA methylation with gene expression assays between KD and healthy controls, the top Gene Ontology items were also involved in leukocyte migration, neutrophil migration, and neutrophil chemotaxis. We further found that specific CpG markers were hypo-methylated in KD compared with controls, which promotes and represses the expression of S100A genes [14]. In an in vitro study, the addition of S100A family proteins can enhance leukocyte transendothelial migration, which suggests that the S100A family plays an important role in the pathogenesis of KD [14]. Accumulating evidence has supported the involvement of neutrophils in the pathogenesis of many autoimmune diseases [27]. Takahashi et al. revealed that activated neutrophils are involved in the early stage of coronary arteritis in KD patients [28]. Neutrophils can be involved in regulating disease development through the matrix metalloproteinase (MMP)-9-mediated degradation of extracellular matrix [29]. Likewise, we have also reported epigenetic hypomethylation, an increased MMP-9 transcript, and the upregulation of MMP-9 in KD patients with CAL formation [30].

CD177 is an expressed human surface marker, and 45–60% of neutrophils in the periphery blood of healthy individuals are CD177+ [31]. CD177 is a heterophilic binding partner of platelet endothelial cell adhesion molecule-1 that participates in neutrophil transmigration [32, 33]. The function of CD177+ neutrophils is to enhance antimicrobial activity, which is associated with the increased production of reactive oxygen species, neutrophil extracellular traps, and bactericidal peptides [27]. One recent study reported that targeting CD177+ neutrophils may be beneficial for treating inflammatory bowel disease [34]. Elevated CD177 mRNA levels and protein expressions have also been found in circulating neutrophils after septic shock [35]. In agreement with our findings, Abe et al. also demonstrated that neutrophils in the acute stage of KD not only increased in number but also activated and expressed a variety of granulocyte-specific genes, including CD177, polycythemia rubra vera 1, and haptoglobin, when compared with febrile controls [36]. Altogether, our findings highlight the epigenetic hypomethylation of the CD177 promoter, as well as the upregulation of CD177 transcript expression. Of particular note, the area under the curve (AUC) values of CD177 between KD patients and controls is 0.937, which indicated that CD177 may be a good marker for KD.

Incomplete KD appears to be more common in infants than in older children, while the laboratory findings of incomplete KD resemble those of classic KD [20]. We observed CD177 to be higher in classic KD than incomplete KD, which may indicate the importance of neutrophil migration, neutrophil chemotaxis, and leukocyte migration in the presentation of clinical KD symptoms.

This study has certain limitations. First, all of our KD patients belonged to the Taiwanese population, so our findings needs to be investigated in other KD populations, and clinical samples of a second population should be considered to confirm our findings. Second, we studied the CD177 expression in peripheral WBC, but studying CD177 expression in infiltrated neutrophils of coronary arteries in early-stage KD patients could yield better results. Such research would provide important insights into the pathophysiology of neutrophils in the CAL of KD, which may ultimately lead to developing novel approaches for treating the disease.

## Conclusions

In short, a better understanding of the fundamental mechanisms of neutrophil activation and migration in KD will improve knowledge of the pathogenesis of KD. Our study is the first to observe DNA hypomethylation and increased CD177 transcripts in KD compared to both types of control subjects. Furthermore, CD177 was related to the typical presentation of KD and associated with IVIG resistance in KD patients.

## Abbreviations

AUC: Area under the curve
CAL: Coronary artery lesion
IVIG: Intravenous immunoglobulin
KD: Kawasaki disease
MMP: Matrix metalloproteinase
